# Antihypertensive Effects of Polyphenolic Extract from Korean Red Pine (*Pinus densiflora* Sieb. et Zucc.) Bark in Spontaneously Hypertensive Rats

**DOI:** 10.3390/antiox9040333

**Published:** 2020-04-19

**Authors:** Kwan Joong Kim, Eun-Sang Hwang, Min-Jeong Kim, Ji-Ho Park, Dae-Ok Kim

**Affiliations:** 1 Graduate School of Biotechnology, Kyung Hee University, Yongin 17104, Korea; Joong@khu.ac.kr; 2 Department of Gerontology, Graduate School of East-West Medical Science, Kyung Hee University, Yongin 17104, Korea; 3 Department of Food Science and Biotechnology, Kyung Hee University, Yongin 17104, Korea; mkimella111@khu.ac.kr; 4 Department of East-West Medicine, Kyung Hee University, Yongin 17104, Korea

**Keywords:** angiotensin-converting enzyme, angiotensin II, blood pressure, phenolics, red pine bark, spontaneously hypertensive rat

## Abstract

Korean red pine (*Pinus densiflora* Sieb. et Zucc.) bark is a by-product of the wood industry and contains a high level of antioxidative phenolics including flavonoids, which have a variety of beneficial health effects. This study aimed to investigate the antihypertensive effects of *P. densiflora* bark extract (Korean red pine bark extract; KRPBE) in spontaneously hypertensive rats (SHRs). A group of Wistar-Kyoto rats as a normotensive group was orally fed tap water. Four groups of SHRs were orally fed tap water, captopril (a positive control), 50 mg/kg/day of KRPBE, and 150 mg/kg/day of KRPBE, respectively. Blood pressure of rats was measured once every week for seven weeks of oral administration. After seven weeks, the lungs, kidneys, and serum were collected from rats, then angiotensin-converting enzyme (ACE) activity, angiotensin II content, and malondialdehyde (MDA) content were determined. Blood pressure of the captopril- and KRPBE-treated groups was significantly lower than that of the SHR control group. The ACE activity, angiotensin II content, and MDA content significantly decreased in the captopril- and KRPBE-treated groups than those in the SHR control group. High-performance liquid chromatography analysis revealed six phenolics in KRPBE: protocatechuic acid, procyanidin B1, catechin, caffeic acid, vanillin, and taxifolin. KRPBE, which contains plenty of antioxidative phenolics, has antihypertensive effects partly due to reduction of ACE activity and angiotensin II content, and its antioxidative effect.

## 1. Introduction

Hypertension is a primary risk factor of cardiovascular disease, which is one of the leading causes of death [1,2]. The renin-angiotensin system (RAS) is a key mechanism in the regulation of blood pressure by producing a potent hypertensive peptide, angiotensin II [3]. In the RAS, angiotensinogen is expressed in the liver, converted to angiotensin I by renin in the circulating blood, and finally converted to angiotensin II by the angiotensin-converting enzyme (ACE) [4]. ACE inhibitors (ACEIs), such as captopril (CAP), ramipril, and enalapril, are frequently used antihypertensives, but these synthetic ACEIs have long-term side effects including retarded wound healing, headache, dizziness, nausea, and kidney damage [5,6,7]. Thus, interest in natural ACEIs such as phenolics recently has increased [8,9].

Reactive oxygen species (ROS) that affect various signaling functions of cells in human metabolism have been regarded as one of factors contributing to hypertension. Several mechanisms through which ROS induce hypertension include decreasing nitric oxide bioavailability [10], up-regulating ACE activity [11], and activating angiotensin II type 1 (AT_1_) receptor [12]. Antihypertensives, such as CAP and enalapril, decreased blood pressure by ACE activity inhibition [13]. Due to their antioxidant properties, some naturally derived polyphenols are known to have antihypertensive effects through inhibiting ACE activity, increasing the bioavailability of nitric oxide, and inhibiting overexpression of AT_1_ receptors [8,9]. The research suggested that scavenging ROS is also important for the treatment of hypertension, and that ACEI such as phenolics with antioxidative capacity is a preferable antihypertensive agent.

*Pinus densiflora* Sieb. et Zucc., or Korean red pine, is a species of pine that is native to Korea, Japan, and Russia [14]. Korean red pine occupies approximately 67% of coniferous forests in Korea [15]. Pine bark accounts for 10–15% of a whole pine tree and is removed for pulp production [14]. Some removed bark is also used for fuel, but a large amount is discarded [14,16]. Given the presence of a wide range of bioactive phenolics including condensed tannins and flavonoids in pine bark, there have been studies on high-value health-promoting products made of abandoned pine bark [14,17].

Previous study demonstrated that various phenolics, such as catechin, taxifolin, protocatechuic acid, and vanillin, are found in Korean red pine bark extract (KRPBE), and exhibited strong antioxidative properties [17]. Taxifolin, a flavanone, and epicatechin, a flavan-3-ol, have been reported to have blood pressure lowering effects due to their antioxidant capacity [18,19]. Procyanidins, which are made using catechin and epicatechin as building blocks, were also reported to have antihypertensive effects [20,21]. However, the antihypertensive effects of KRPBE have not been studied.

In this study, we evaluated the effect of long-term intake of KRPBE on blood pressure in spontaneously hypertensive rats (SHRs). Furthermore, we investigated the target mechanism of KRPBE on RAS by measuring ACE activity, angiotensin II content, and malondialdehyde (MDA) content in the lungs, serum, and kidneys. We performed high-performance liquid chromatography (HPLC) analysis to identify substances that could be responsible for the antihypertensive effects of KRPBE.

## 2. Materials and Methods

### 2.1. Chemicals

KRPBE was extracted using water and obtained from Nutrapharm Ltd. (Yongin, Korea). Bovine serum albumin, potassium phosphate dibasic, CAP, 1,1,3,3-tetramethoxypropane, 2-thiobarbituric acid, trichloroacetic acid, *o*-phthaldialdehyde, *N*-hippuryl-His-Leu hydrate (HHL), histidylleucine (His-Leu; HL), catechin, taxifolin, vanillin, protocatechuic acid, and caffeic acid were purchased from Sigma-Aldrich Co., LLC (St. Louis, MO, USA). Procyanidin B1 was purchased from Extrasynthese (Genay, France). Ethyl ether was purchased from Samchun Chemical Co., Ltd. (Seoul, Korea). All other reagents used were of analytical or HPLC grade.

### 2.2. Quantification of Phenolics Using HPLC

Phenolics in KRPBE were quantitatively analyzed using a reversed-phase HPLC system (Agilent 1200; Agilent Technologies, Santa Clara, CA, USA) equipped with an autosampler, a diode array detector, and a degasser. A reversed-phase column (250 × 4.6 mm, 5 μm; Agilent Zorbax Eclipse XDB-C18; Agilent Technologies) was used. The injection volume was 5 μL. The flow rate was 0.8 mL/min. The two mobile phases were water with 0.1% (*v/v*) formic acid (solvent A) and acetonitrile with 0.1 (*v/v*) formic acid (solvent B). The gradient elution profile was as follows: 95% A/5% B at 0 min, 85% A/15% B at 25 min, 65% A/35% B at 45 min, 30% A/70% B at 50 min, 20% A/80% B at 58 min, 95% A/5% B at 60 min, and 95% A/5% B at 65 min. The wavelengths for detection were set at 320 nm for caffeic acid and 280 nm for protocatechuic acid, procyanidin B1, catechin, vanillin, and taxifolin. Phenolics in KRPBE were identified by comparison of UV–visible spectra, retention times, and spiked inputs with commercial standards. Phenolics were quantified using calibration curves that relate different concentrations of authentic standards to the areas of their corresponding peaks. 

### 2.3. Animals 

Twenty four four-week-old male SHRs (approximately 200 g each) and six age-matched male Wistar-Kyoto rats (WKRs) were purchased from Orient Bio Inc. (Sungnam, Korea). Rats were housed in a laboratory cage under controlled conditions: temperature of 23 °C, 56% relative humidity, and a 12 h light-dark cycle from 8 a.m. to 8 p.m. Rats had access to standard diet (5L79; Orient Bio Inc.; Sungnam, Korea) and water *ad libitum* throughout the experiment period. All animal procedures complied with the Institutional Animal Care and Use Committee of Kyung Hee University with approval number: KHUASP (SE)-17-019 (approval date: 12 June 2017) and were performed in accordance with the guiding principles for the care and use of animals approved by the Council of the National Institutes of Health Guide for the Care and Use of Laboratory Animals.

### 2.4. Oral Administration of KRPBE 

After one week of adaptation, the five-week-old SHRs were divided into four groups consisting of six rats. These four groups were each randomly assigned to a control group, a positive control group, and two KRPBE-treated groups. Tap water (WKR group, a normotensive group, and SHR group, a control group), 15 mg/kg body weight/day of CAP (SHR + CAP group, a positive control group), 50 mg/kg body weight/day of KRPBE (SHR + KRPBE50 group), and 150 mg/kg body weight/day of KRPBE (SHR + KRPBE150 group) were orally administered to rats for seven weeks each day at 9 a.m. 

### 2.5. Measurement of Blood Pressure

Systolic blood pressure (SBP) and diastolic blood pressure (DBP) were measured noninvasively using the CODA® tail-cuff blood pressure system (Kent Scientific Corp., Torrington, CT, USA) once a week at the same time of the day. The rats were kept at 37 °C for 15 min in a black acryl animal holder before measuring blood pressure to intensify the pulsation of the tail artery and minimize stress. 

### 2.6. Collection of Tissue and Serum

After seven weeks of oral administration of tap water, CAP, and KRPBE, the 12-week-old experimental animals were sacrificed. The animals were anesthetized with ethyl ether, and then their lungs and kidneys were rapidly harvested. The lungs and kidneys were homogenized with lysis buffer. The homogenized lungs and kidneys in the lysis buffer were sonicated (NRE-02; Next Advance, Troy, NY, USA) and centrifuged at 18,403× *g* for 20 min at 4 °C (PK121R; Alc International S.R.L., Cologno Monzese, Italy). The supernatant was stored at −80 °C prior to analysis. Blood was collected in a serum-separating tube (BD Vacutainer™ SST™ II Advance Tubes; Thermo Fisher Scientific, Waltham, MA, USA) with an anticoagulant and centrifuged at 18,403× *g* for 20 min at 4 °C. Aliquots of serum were stored at −80 °C prior to analysis.

### 2.7. Measurement of ACE Activity 

ACE activities of serum, lungs, and kidneys were measured according to the modified method described by Schwager et al. [22]. In brief, 30 μL of 5.7 mM HHL was injected into a 96-well plate and then incubated for 15 min at 37 °C. After incubation, 3 μL of appropriately diluted tissue lysate, serum, or HL standard was mixed with HHL, which was incubated for 25 min at 37 °C. To stop the reaction between HHL and the sample (or HL standard), 177 μL of 0.28 M NaOH solution was added. Fifteen microliters of *o*-phthaldialdehyde (20 mg/mL) were immediately added to the wells followed by shaking for 10 min at 25 °C. Then, 25 μL of 3 M HCl was added to each well to stop the reaction between samples and *o*-phthaldialdehyde. Fluorescence was measured at excitation 360 nm and emission 485 nm using a microplate reader (Victor X3; PerkinElmer Inc., Waltham, MA, USA). ACE activity (μM/min/mg enzyme) was determined using an HL standard calibration curve. 

### 2.8. Measurement of Angiotensin II Content 

An angiotensin II ELISA kit was purchased from Antibodies-Online GmbH (ABIN1558956; Aachen, Germany). Levels of angiotensin II in the lungs, kidneys, and serum were measured with a microplate reader (Victor X3; PerkinElmer Inc.) according to the manufacturer’s instructions. 

### 2.9. Measurement of MDA Content

Lipid peroxidation of serum and tissue (lungs and kidneys) was determined according to the modified method described by Draper et al. [23]. In brief, 100 μL of appropriately diluted tissue lysate or serum was mixed with 200 μL of 10% (*v/v*) trichloroacetic acid, and then incubated on ice for 10 min to facilitate protein sedimentation. The reactive supernatant of acidified tissue lysate or serum was moved to other microtubes, and 200 μL of the supernatant or 1,1,3,3-tetramethoxypropane standard was mixed with 200 μL of 0.67% (*w/v*) thiobarbituric acid and then heated for 10 min at 100 °C. After the heat reaction, the reactant was cooled, moved to a 96-well plate, and measured at 531 nm using a microplate reader (Victor X3; PerkinElmer Inc.). MDA content of tissue lysate (nM MDA/mg protein of tissue lysate) or serum (nM MDA/mL of serum) was determined using a 1,1,3,3-tetramethoxypropane standard calibration curve. 

### 2.10. Statistical Analysis 

All data were expressed as the mean ± standard error of the mean (*n* = 6). Statistical analysis was performed using SPSS software (Version 23.0; IBM SPSS Statistics Inc., Chicago, IL, USA). One-way analysis of variance was performed to evaluate the differences in mean values. Significant differences were verified by the Tukey-Kramer honestly significant difference test (*p* < 0.05) and significant levels are represented as asterisks and hashtags (* *p* < 0.05, ** *p* < 0.01, *** *p* < 0.001 vs. WKR and # *p* < 0.05, ## *p* < 0.01, ### *p* < 0.001 vs. SHR).

## 3. Results

### 3.1. Quantification of Phenolics Using HPLC

Concentrations of six major phenolics (protocatechuic acid, procyanidin B1, catechin, caffeic acid, vanillin, and taxifolin) in KRPBE are presented in Table 1. Concentrations of these six major phenolics in KRPBE decreased as follows: procyanidin B1 > catechin > taxifolin > protocatechuic acid > vanillin > caffeic acid. The elution order (retention time) for the six phenolics identified using reversed-phase HPLC was as follows: protocatechuic acid (14.1 min) > procyanidin B1 (18.8 min) > catechin (22.7 min) > caffeic acid (26.1 min) > vanillin (32.2 min) > taxifolin (38.3 min) (Figure S1).

### 3.2. Effect of KRPBE on Blood Pressure in WKRs and SHRs

**Figure 1:**
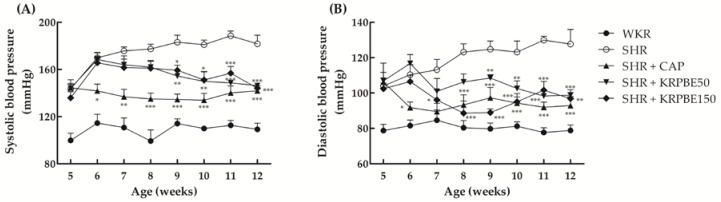
Systolic blood pressure (SBP) (**A**) and diastolic blood pressure (DBP) (**B**) of Wistar-Kyoto rats (WKRs) and spontaneously hypertensive rats (SHRs) during seven weeks of oral administration. WKR group (tap water), SHR group (tap water), SHR + captopril (CAP) group (15 mg/kg body weight/day of CAP), SHR + Korean red pine bark extract (KRPBE)50 group (50 mg/kg body weight/day of KRPBE), and SHR + KRPBE150 group (150 mg/kg body weight/day of KRPBE) were orally administrated for seven weeks. Each group consists of six rats. Tukey–Kramer honestly significant difference test: * *p* < 0.05, ** *p* < 0.01, *** *p* < 0.001 vs. SHR.

Changes in SBP and DBP of WKR group (normotensive control), SHR group (control group), SHR + CAP group (positive control), SHR + KRPBE50 group, and SHR + KRPBE150 group for the seven-week administration are shown in Figure 1. There were no significant differences in SBP among any of the SHR groups at the beginning of the study (Figure 1A). In the seventh week of daily oral administration (12-week-old), the SHR + CAP, SHR + KRPBE50, and SHR + KRPBE150 groups had mean SBPs that were approximately 78%, 81%, and 79% of that of the SHR control group, respectively (Figure 1A). Significant decreases in SBP in the SHR + KRPBE50 and SHR + KRPBE150 groups were observed after four weeks of daily oral administration (9-week-old) of the 50 and 150 mg/kg body weight of KRPBE, respectively (Figure 1A).

There were no significant differences in DBP between all but the WKR group at the beginning of the study period (Figure 1B). The SHR + CAP group had significantly lowered DBPs than the SHR control group after the first week of administration (six-week-old), whereas the SHR + KRPBE50 and SHR + KRPBE150 groups had significantly lowered DBPs than the SHR control group after the fourth (nine-week-old) and second (seven-week-old) week of administration, respectively (Figure 1B). In the seventh week of oral administration (12-week-old), the SHR + CAP, SHR + KRPBE50, and SHR + KRPBE150 groups had mean DBPs that were 73%, 77%, and 76% of that of the SHR control group, respectively (Figure 1B).

### 3.3. ACE Activity in the Lungs, Kidneys, and Serum of WKRs and SHRs

**Figure 2:**
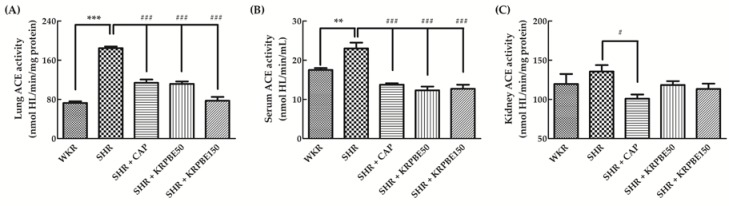
Angiotensin-converting enzyme (ACE) activities in lungs (**A**), serum (**B**), and kidneys (**C**) of WKRs and SHRs after seven weeks of oral administration. WKR group (tap water), SHR group (tap water), SHR + CAP group (15 mg/kg body weight/day of CAP), SHR + KRPBE50 group (50 mg/kg body weight/day of KRPBE), and SHR + KRPBE150 group (150 mg/kg body weight/day of KRPBE) were orally administrated for seven weeks. ACE activity was determined using His-Leu (HL) standard calibration curve. Each group consists of six rats. Tukey–Kramer honestly significant difference test: ** *p* < 0.01, *** *p* < 0.001 vs. WKR and # *p* < 0.05, ### *p* < 0.001 vs. SHR.

ACE activity in the lungs, kidneys, and serum from SHRs and WKRs was measured after sacrifice of the rats used in this study (Figure 2). The SHR control group showed significantly (*p* < 0.001) higher lung ACE activity than the WKR group (Figure 2A). The SHR + CAP, SHR + KRPBE50, and SHR + KRPBE150 groups showed significantly (*p* < 0.001) lower lung ACE activity than that of the SHR control group (61.7%, 60.5%, and 42.0%, respectively). In particular, the SHR + KRPBE150 group had a similar level of lung ACE activity as the WKR group (39.4% of that in the SHR control group). 

The SHR control group showed significantly (*p* < 0.01) higher serum ACE activity than the WKR group (Figure 2B). The SHR + CAP, SHR + KRPBE50, and SHR + KRPBE150 groups showed significantly (*p* < 0.001) lower levels of serum ACE activity than the SHR control group (60.0%, 53.6%, and 55.2%, respectively). The SHR + CAP, SHR + KRPBE50, and SHR + KRPBE150 groups had lower serum ACE activity than the WKR group, which had approximately 76.1% of that of the SHR control group (Figure 2B). 

Kidney ACE activity was not significantly different between the SHR and WKR groups (Figure 2C). The two groups treated with KRPBE had no difference in kidney ACE activity compared to the SHR control group. However, the SHR + CAP group showed significantly (*p* < 0.05) lower kidney ACE activity (74.5%) than that of the SHR control group.

### 3.4. Angiotensin II Content in Lungs, Kidneys, and Serum of WKRs and SHRs

**Figure 3:**
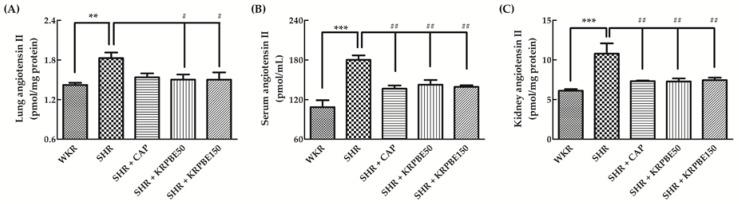
Angiotensin II content in lungs (**A**), serum (**B**), and kidneys (**C**) of WKRs and SHRs after seven weeks of oral administration. WKR group (tap water), SHR group (tap water), SHR + CAP group (15 mg/kg body weight/day of CAP), SHR + KRPBE50 group (50 mg/kg body weight/day of KRPBE), and SHR + KRPBE150 group (150 mg/kg body weight/day of KRPBE) were orally administrated for seven weeks. Each group consists of six rats. Tukey-Kramer honestly significant difference test: ** *p* < 0.01, *** *p* < 0.001 vs. WKR and # *p* < 0.05, ## *p* < 0.01 vs. SHR.

We evaluated angiotensin II content in the lungs, kidneys, and serum from SHRs and WKRs after seven weeks of blood pressure measurements (Figure 3). The SHR control group showed significantly (*p* < 0.01) higher lung angiotensin II content than the WKR group (Figure 3A). The SHR + CAP group had no difference in lung angiotensin II content compared to the SHR control group, whereas the SHR + KRPBE50 and SHR + KRPBE150 groups showed significantly (*p* < 0.05) lower lung angiotensin II content than the SHR control group (82.2% and 82.1%, respectively) (Figure 3A). 

The SHR control group showed significantly (*p* < 0.001) higher serum angiotensin II content than the WKR group (Figure 3B). The SHR + CAP, SHR + KRPBE50, and SHR + KRPBE150 groups showed significantly (*p* < 0.01) lower serum angiotensin II content than the SHR control group (75.7%, 79.1%, and 77.2%, respectively) (Figure 3B). 

The SHR control group showed significantly (*p* < 0.001) higher kidney angiotensin II content than the WKR group (Figure 3C). The SHR + CAP, SHR + KRPBE50, and SHR + KRPBE150 groups showed significantly (*p* < 0.01) lower kidney angiotensin II content than the SHR control group (67.7%, 67.5%, and 69.1%, respectively) (Figure 3C). 

### 3.5. MDA Content in Lungs, Kidneys, and Serum of WKRs and SHRs

**Figure 4:**
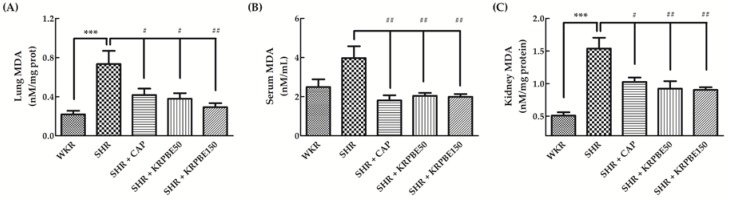
Malondialdehyde (MDA) content in lungs (**A**), serum (**B**), and kidneys (**C**) of WKRs and SHRs after seven weeks of oral administration. WKR group (tap water), SHR group (tap water), SHR + CAP group (15 mg/kg body weight/day of CAP), SHR + KRPBE50 group (50 mg/kg body weight/day of KRPBE), and SHR + KRPBE150 group (150 mg/kg body weight/day of KRPBE) were orally administrated for seven weeks. Each group consists of six rats. Tukey-Kramer honestly significant difference test: *** *p* < 0.001 vs. WKR and # *p* < 0.05, ## *p* < 0.01 vs. SHR.

Total MDA content of the lungs, kidneys, and serum from SHRs and WKRs are shown in Figure 4. The SHR control group showed significantly (*p* < 0.01) higher total lung MDA content than the WKR group. The SHR + CAP, SHR + KRPBE50, and SHR + KRPBE150 groups had significantly (*p* < 0.05 or *p* < 0.01) lower total MDA content than the SHR control group (56.8%, 51.7%, and 40.0%, respectively) (Figure 4A). 

The total serum MDA content was not significantly different between the SHR and WKR group (Figure 4B). However, the SHR + CAP, SHR + KRPBE50, and SHR + KRPBE150 groups had significantly (*p* < 0.01) lower total MDA content than the SHR control group (45.5%, 51.5%, and 50.1%, respectively) (Figure 4B). 

The SHR control group showed significantly (*p* < 0.001) higher total kidney MDA content than the WKR group (Figure 4C). The SHR + CAP, SHR + KRPBE50, and SHR + KRPBE150 groups showed significantly (*p* < 0.05 or *p* < 0.01) lower total MDA content than the SHR control group (66.6%, 59.9%, and 58.8%, respectively) (Figure 4C). 

## 4. Discussion

SHR is the most widely used in vivo model for human essential hypertension studies [24]. The SHR strain was produced by selective inbreeding of WKR with high blood pressure, shows high blood pressure after five weeks, and tends to keep increasing blood pressure with age [24]. Pathophysiology of hypertension in SHR is due to overall changes in RAS components, such as renin activity, ACE activity, angiotensin II content, AT_1_ receptor expression, and NADPH oxidase activity [12,25,26]. Sodium imbalance in SHR causes the inappropriate release and activation of RAS components leading to overexpression of AT_1_ receptor and angiotensinogen and the increase in renin and ACE activities [12,25,26]. The overexpression of AT_1_ receptor will increase NADPH oxidase activity, which produces ROS, which then damages tissues such as the endothelial aorta and kidney in the SHR [25]. Phenolics, such as flavonoids, with ACE inhibition and antioxidant capacity have been studied recently [8,9,27]. In this study, the antihypertensive effects of KRPBE, which includes antioxidative phenolics, were evaluated in relation to ACE activity, angiotensin II content, and oxidative damage of RAS-related organs.

Angiotensinogen is released in the liver and then hydrolyzed to angiotensin I by the action of renin, which is secreted from the kidneys. ACE, the key component of RAS, is released in various organs, such as the lungs and kidneys [4,28]. ACE cleaves two C-terminal residues (His-Leu) of the inactive decapeptide angiotensin I to produce the octapeptide vasoconstrictor, angiotensin II [22]. Angiotensin II affects the central nervous system by increasing the secretion of vasopressins, such as antidiuretic hormone, arginine vasopressin, and argipressin, which all cause high blood pressure [29]. Angiotensin II also stimulates smooth muscle contraction and the sodium-hydrogen antiporter to promote sodium reabsorption and hydrogen secretion, causing high blood pressure [28,29]. Angiotensin II reduction by decreasing ACE activity is regarded as the most important blood pressure control mechanism in RAS [26,27]. Thus, ACEIs are mainly used to treat hypertension [26].

The lungs of SHR are known to have high ACE content and specific ACE activity [30]. Angiotensin II has stronger vasoconstrictive effects in the kidneys than in other organs such as the lungs, brain, and blood vessel walls because all components of RAS exist within the kidneys, although the ACE content and activity are low [28,31]. Therefore, it is important to evaluate the RAS mediators of blood pressure in both the lungs and kidneys. Furthermore, observing the overall flow of RAS components, such as ACE and angiotensin II, through the blood is also important to explain the blood pressure lowering effects of KRPBE.

In our study, two SHR groups fed with KRPBE (SHR + KRPBE50 and SHR + KRPBE150) had significantly lower ACE activity in the lungs and serum and had also lower angiotensin II content in the lungs, kidneys, and serum compared to the SHR control group, which is consistent with a previous study reporting that angiotensin II content is dependent on ACE activity [12]. The ACE activity in the kidneys of the SHR groups fed with KRPBE was not significantly lower than in the SHR control group, but the angiotensin II content in the SHR groups fed with KRPBE were significantly lower than in the SHR control group. Rat kidneys were reported to have relatively lower specific ACE activity because the enzyme may be localized to other organs such as afferent arterioles [30]. Thus, ACE activity was not significantly different among kidneys from the rats used in this study. These results suggest that regulated ACE activity and angiotensin II content would be one of the mechanisms of blood pressure lowering effects of KRPBE.

MDA is an end-product of lipid peroxidation, which is accelerated by ROS, one cause of cellular damage [32]. Damage in the kidneys from excessive ROS results in hypertension due to a malfunction in sodium reabsorption [29], upregulated ACE expression [11], and increased angiotensin II generation [12,33]. Several studies focused on changes of the indicators, such as lipid peroxidation of tissue, NADPH oxidase activity, and antioxidant enzyme activity, of oxidative stress accompanied by down-regulating RAS in the hypertensive model [4,5,12]. It has been reported that angiotensin II upregulates AT_1_ receptor expression by inducing NADPH oxidase-dependent oxidative stress, but antioxidant such as superoxide dismutase mimetic compound tempol (4-hydroxy-2,2,6,6-tetramethypiperidine-*N*-oxyl) inhibits a positive feedback pathway for angiotensin II to stimulated AT_1_ receptor expression [25]. Our results showed reduced MDA levels in the lungs, serum, and kidneys of SHRs after KRPBE intake, suggesting that antihypertensive effects of KRPBE are partly due to not only direct improvement of excessive ROS, but also inhibition of positive feedback loop for RAS by its antioxidative phenolics.

Six major phenolics were identified in KRPBE. It has been reported that *P. densiflora* bark contains polyphenols, such as procyanidin B1, catechin, and taxifolin [17,34]. Many flavonoids have high antioxidant capacity due to the presence of hydroxyl groups on the flavonoid backbone and inhibit production of ROS, which leads to increased bioavailability of nitric oxide, a vasodilator [10,19,35,36]. In addition, flavonoids have been reported to upregulate the expression of endothelial nitric oxide synthase, which helps reduce blood pressure [10,36]. Flavonoids inhibit ACE activity by chelating zinc ion on ACE, thereby flavonoids work as ACEIs. Catechin and taxifolin, which were found in KRPBE in this study, have been reported to have antioxidant capacity [35], and thus lower blood pressure [18,37]. Furthermore, condensed tannins, such as procyanidin B1, the most dominant compound in KRPBE, have been reported to show ACE-inhibitory effects in reducing blood pressure [20,21]. Hence, the blood pressure lowering effects of KRPBE may be attributed to its various bioactive phenolics and their ACE inhibitory and antioxidant activities.

Synthetic ACEIs such as CAP and ramipril are widely used at various stages of hypertension, but they have several side effects [5,6]. In particular, many patients taking synthetic ACEIs have been suffering from adverse effects such as delay of wound healing, which results in skin eruptions including pemphigus vulgaris acantholysis [7,38]. CAP down-regulated the expression of the insulin-like growth factor receptor and phosphorylated mitogen-activated protein kinases, resulting in decreased collagen synthesis [39]. In addition, ramipril has been reported to increase the expression of caspase 3 in fibroblasts in a 3D wound model and adversely affects wound healing [40]. In contrast, many phenolics with antioxidant capacity have been known to promote wound healing by regulating the inflammatory stage of the wound healing process by scavenging ROS [41]. It was reported that 60% ethanol/water extraction of *P. densiflora* bark regulated wound healing-involved cytokines such as interleukin (IL)-4, IL-5, and tumor necrosis factor alpha, resulting in attenuated atopic dermatitis. Pycnogenol®, which has the similar phytochemical composition to KRPBE such as procyanidin B1 and catechin, reduced the wound healing time of Sprague-Dawley rats in a dose-dependent manner and significantly reduced scar sizes [42]. In the study of Cetin et al. [43], *P. brutia* extract promoted wound healing by regulating the antioxidant status such as superoxide dismutase, catalase, and MDA content in albino rats. In addition, procyanidin B1, a main polyphenol in KRPBE, was previously reported to inhibit collagenase and elastase, which regulates matrix metalloproteinase and extracellular proteinase balance [44]. Catechin increased the thermal stability of collagen and increased resistance to structure-destabilizing agent, urea, through hydrogen bonding and hydrophobic interaction with collagen [45]. In our previous [17] and present results, KRPBE and its phenolics had antioxidant, suggesting that KRPBE has the potential in regulating inflammation in wound healing processes [41]. Many published studies and our results suggest that KRPBE had an antihypertensive effect by ACE inhibition, but it anticipates to have fewer side effects such as delay of wound healing than synthetic ACEIs.

## 5. Conclusions

Oral administration of KRPBE reduced the blood pressure of SHRs. Lungs, kidneys, and serum of SHRs fed with KRPBE showed inhibited ACE activity, reduced angiotensin II content, and decreased lipid peroxidation. These antihypertensive effects may be due to the antioxidant capacity and ACE inhibition of various phenolics, such as protocatechuic acid, procyanidin B1, catechin, caffeic acid, vanillin, and taxifolin, in KRPBE. Our study suggests that KRPBE rich in antioxidative phenolics can be used as a new functional food ingredient for lowering hypertension by regulating RAS components and inhibiting lipid peroxidation. In addition, hypertension is associated with a variety of mechanisms, such as ACE inhibition in RAS, nitric oxide bioavailability, and regulation of endothelium-dependent relaxation. Therefore, further study on a clinical trial and other antihypertensive mechanisms of KRPBE is needed.

## Figures and Tables

**Table 1:** Concentrations of phenolics in Korean red pine (*Pinus densiflora* Sieb. et Zucc.) bark extract measured using reversed-phase high-performance liquid chromatography.

Phenolics	Concentration (mg/g Dry Weight)
Protocatechuic acid	3.99 ± 0.21
Procyanidin B1	23.78 ± 1.17
Catechin	9.06 ± 0.42
Caffeic acid	0.29 ± 0.01
Vanillin	0.41 ± 0.01
Taxifolin	6.38 ± 0.29

## Supplementary Materials

The following are available online at https://www.mdpi.com/2076-3921/9/4/333/s1, Figure S1. High-performance liquid chromatography traces of mixture of standards at 280 nm (**A**) and 320 nm (**B**), and Korean red pine (*Pinus densiflora* Sieb. et Zucc.) bark extract at 280 nm (**C**) and 320 nm (**D**).

## Abbreviations

ACE	angiotensin-converting enzyme
ACEI	angiotensin-converting enzyme inhibitor
AT_1_	angiotensin II type 1
CAP	captopril
DBP	diastolic blood pressure
HHL	***N***-hippuryl-His-Leu hydrate
HL	His-Leu
HPLC	high-performance liquid chromatography
IL	interleukin
KRPBE	Korean red pine bark extract
MDA	malondialdehyde
RAS	renin-angiotensin system
ROS	reactive oxygen species
SBP	systolic blood pressure
SHR	spontaneously hypertensive rat
WKR	Wistar-Kyoto rat
