# Supplementary material for: Antihypertensive Effects of Polyphenolic Extract from Korean Red Pine (Pinus densiflora Sieb. et Zucc.) Bark in Spontaneously Hypertensive Rats
Source: Antioxidants (Basel). 2020 Apr 19;9(4):333. doi: 10.3390/antiox9040333 (PMC7222369; doi:10.3390/antiox9040333)
Supplement: Supplementary file 1 [file antioxidants-09-00333-s001.pdf]

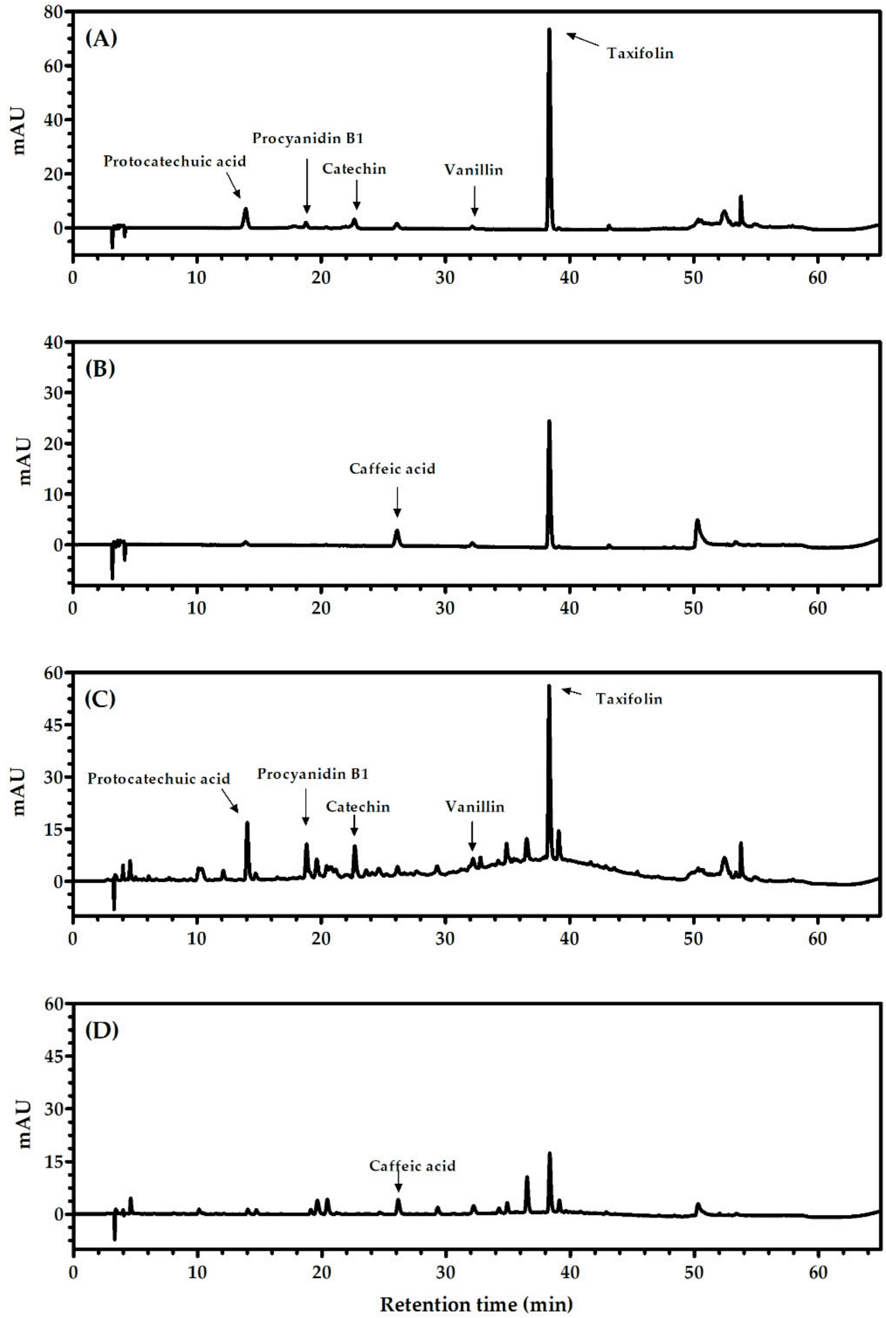

Supplementary Figure S1. High-performance liquid chromatography traces of mixture of standards at 280 nm (A) and 320 nm (B), and Korean red pine (*Pinus densiflora* Sieb. et Zucc.) bark extract at 280 nm (C) and 320 nm (D).
